# Hypertension among adults enrolled in HIV care in northern Tanzania: comorbidities, cardiovascular risk, and knowledge, attitudes and practices

**DOI:** 10.11604/pamj.2022.41.285.26952

**Published:** 2022-04-07

**Authors:** Preeti Manavalan, Deng Buok Madut, Julian Thornton Hertz, Nathan Maclyn Thielman, Nwora Lance Okeke, Blandina Theophil Mmbaga, Melissa Harper Watt

**Affiliations:** 1 Division of Infectious Diseases and Global Medicine, University of Florida, Gainesville FL, USA,; 2 Division of Infectious Diseases, Duke University, Durham, NC, USA,; 3 Duke Global Health Institute, Durham, NC, USA,; 4 Division of Emergency Medicine, Duke University, Durham, NC, USA,; 5 Kilimanjaro Clinical Research Institute, Moshi, Tanzania,; 6 Kilimanjaro Christian Medical University College, Moshi, Tanzania,; 7 Department of Population Health Sciences, University of Utah, Salt Lake City, UT, USA

**Keywords:** Tanzania, sub-Saharan Africa, hypertension, non-communicable diseases, atherosclerotic cardiovascular disease risk

## Abstract

**Cite this article:**

Preeti Manavalan et al. Hypertension among adults enrolled in HIV care in northern Tanzania: comorbidities, cardiovascular risk, and knowledge, attitudes and practices. Pan African Medical Journal. 2022;41(285). 10.11604/pamj.2022.41.285.26952

**Introduction:**

the epidemiology of non-communicable diseases (NCDs) among people living with HIV (PLHIV) in sub-Saharan Africa is poorly described. In this observational study we examined a cohort of hypertensive PLHIV in northern Tanzania and described comorbidities, cardiovascular risk, and hypertension knowledge, attitudes and practices.

**Methods:**

consecutive patients attending an HIV clinic were screened for hypertension; those who met hypertension study criteria were enrolled. Participants completed a hypertension knowledge, attitudes and practices survey, and underwent height, weight, and waist circumference measurements and urine dipstick, fasting blood sugar, and lipid panel analyses. Kidney disease was defined as 1+ proteinuria, diabetes mellitus was defined as fasting glucose >126mg/dL, and 10-year atherosclerotic cardiovascular disease (ASCVD) risk was defined per the Pooled Cohorts Equations.

**Results:**

of 555 screened patients, 105 met hypertension criteria and 91 (86.7%) were enrolled. The prevalence of diabetes mellitus, kidney disease, and overweight or obesity was 8.8%, 28.6%, and 86.7%, respectively. Almost all participants (n=86, 94.5%) had two or more medical comorbidities. More than half (n=39, 52.7%) had intermediate or high 10-year risk for an ASCVD event. While only 3 (3.3%) participants were able to define hypertension correctly, most would seek care at a medical facility (n=89, 97.8%) and take medication chronically for hypertension (n=79, 87.8%).

**Conclusion:**

we found a high burden of medical comorbidity and ASCVD risk among hypertensive PLHIV in northern Tanzania. Integration of routine NCD screening in the HIV clinical setting, in combination with large-scale educational campaigns, has the potential to impact clinical outcomes in this high-risk population.

## Introduction

People living with HIV (PLHIV) who are engaged in HIV care have a prevalence of hypertension, approaching 30% in sub-Saharan Africa (SSA) [1], which may significantly increase their risk of cardiovascular disease-related morbidity and mortality [2, 3]. Despite this high prevalence, rates of hypertension diagnosis, treatment and control in SSA are low [4, 5]. Reasons for poor hypertension outcomes among PLHIV in SSA are not well understood, but likely include inadequate hypertension knowledge, siloed health systems, lack of access to medications and blood pressure equipment, and high costs of care [6-9]. PLHIV who are hypertensive may be at increased risk for other non-communicable diseases (NCDs), including diabetes mellitus and chronic kidney disease, further heightening their risk of serious cardiovascular events [5, 10]. However, the burden of NCDs and cardiovascular disease risk among PLHIV in SSA have not been well-described [11], and clinic-based studies are needed to further define the true burden of comorbidity among this population. Understanding the epidemiology of chronic diseases associated with hypertension as well as patient knowledge, attitudes and practices are critical first steps for developing interventions to reduce cardiovascular related morbidity and mortality among PLHIV. This study examined a cohort of hypertensive patients enrolled in HIV care in northern Tanzania. Using surveys and laboratory investigations, we sought to describe the risk factors, comorbidities, and atherosclerotic cardiovascular disease (ASCVD) risk associated with hypertension and characterize patient knowledge, attitudes and practices towards hypertension.

## Methods

**Study setting:** this study was conducted from October 16, 2018 to December 14, 2018 in the town of Moshi in the Kilimanjaro Region of northern Tanzania at a single HIV clinic located in a government health facility that serves approximately 1200 adults (900 women and 300 men) with HIV per year.

### Study procedures

During routine HIV appointments, all patients attending the HIV clinic were introduced to the study nurse by a member of the clinic staff and were invited to have their blood pressure measured. We have previously described the detailed procedures and findings from these blood pressure assessments elsewhere [4]. Briefly, blood pressure was measured twice in the right arm with at least a five-minute interval between measures using an FDA-approved automatic blood pressure monitor (Omron Healthcare, Bannockburn, Illinois, USA). If at least one of the two measurements was > 140 mmHg systolic or > 90 mmHg diastolic, then the participant was invited to return to the clinic within one to two weeks for repeat blood pressure measurements. The study definition for hypertension included meeting any of the following criteria: 1) a self-reported diagnosis of hypertension, 2) a single blood pressure measurement > 160 mmHg systolic or > 100 mmHg diastolic, or 3) two measurements at separate visits obtained at least one week apart > 140 mmHg systolic or > 90 mmHg diastolic. Individuals were eligible to participate in the study if they met study criteria for hypertension, were over 18 years of age, not pregnant, and seeking HIV care at the study site. During the study period 555 patients were screened for hypertension, of which 105 (19.6%) met study criteria for hypertension and were thus eligible to participate in the study [4]. Eligible participants were invited to complete a knowledge, attitudes and practices (KAP) survey that also included questions about hypertension risk factors and medical comorbidities. The survey was orally administered by the study nurse in Swahili. Participants also completed anthropometric and laboratory investigations including height, weight, waist circumference, urinalysis, fasting glucose finger stick and a lipid panel.

**Survey development**: content from the KAP survey was derived from multiple existing KAP surveys related to hypertension, chronic kidney disease, and cardiovascular disease from SSA [12-15]. Candidate questions, including questions from existing surveys, were written, selected and reviewed by an interdisciplinary team of physicians, scientists and lay people from Tanzania and the US. The survey was translated from English to Swahili and then independently back-translated to confirm fidelity to the content. The adapted KAP survey was piloted with 4 Tanzanians, some with and some without medical backgrounds, to ensure content clarity and fidelity. The final KAP survey consisted of 6 questions regarding hypertension knowledge, 11 questions regarding hypertension attitudes, and 12 questions regarding hypertension practices. Responses to open-ended questions were graded as correct based on independent review by two physicians. If the two reviewers could not come to an agreement, a third physician researcher served as a tiebreaker.

### Study definitions and measures

**Hypertension risk factors and characteristics**: risk factors for hypertension, including dietary intake of fruits, vegetables and salt, use of alcohol and tobacco, family history of hypertension and physical activity, were self-reported. Vigorous physical activity was defined as engaging in 75 minutes or more of vigorous-intensity aerobic activity per week and moderate physical activity was defined as engaging in 150 minutes or more of moderate-intensity aerobic activity per week per the Centre of Disease Control guidelines [16]. High risk alcohol use was defined as more than 3 drinks per day or more than 7 drinks per week for women and more than 4 drinks per day or more than 14 drinks per week for men per the National Institute of Alcohol Abuse and Alcoholism guidelines [17]. Blood pressure control was defined if all blood pressure measurements obtained by the study nurse were < 140 mmHg systolic and < 90 mmHg diastolic.

**Atherosclerotic cardiovascular disease (ASCVD) risk**: ASCVD risk, which measures 10-year risk of a major cardiovascular event for adults 40 years and older, was estimated using the American Heart Association (AHA) and American College of Cardiology (ACC) ASCVD Risk Estimator based on the Pooled Cohort Equations. The Pooled Cohort Equations were developed from sex and race-specific proportional-hazard models. Coefficients of age, sex, systolic blood pressure, antihypertensive drug use, diabetes mellitus, smoking history, HDL cholesterol and total cholesterol are included in the equation models for calculating an estimate of 10-year ASCVD risk. For example, as outlined in the AHA and ACC Guidelines on the Assessment of Cardiovascular Risk, the estimated 10-year risk of an ASCVD event is formally calculated as 1 minus the survival rate at 10 years, raised to the power of the exponent of the coefficient x value sum minus the race and sex-specific overall mean coefficient x value sum (i.e. 1 - S_10_^(lndX´B-MeanX´B)^) [18-20]. The study nurse or the clinic phlebotomist obtained a lipid panel which included total cholesterol, HDL cholesterol and triglycerides. If a participant was less than 40 years of age, they were excluded from the ASCVD risk calculation. Patients were categorized as having a low ASCVD risk if they had < 5.0% risk of a cardiovascular event within 10 years, borderline risk if they had a risk between 5.0% and 7.4%, intermediate risk if they had a risk between 7.5% and 19.9%, and high ASCVD risk if their calculated risk was > 20.0% per AHA and ACC guidelines [20].

**Diabetes mellitus and prediabetes:** the study nurse obtained a fasting blood sugar on all participants using a Bayer Contour glucometer and test strips (Ascensia Diabetes Care, Parsippany, New Jersey, USA). Diabetes mellitus was defined as a fasting blood sugar > 126 mg/dL. Prediabetes was defined as having a fasting blood sugar > 100 and < 126 mg/dL. If a participant did not fast for at least 8 hours prior to testing, prediabetes was defined as a random blood sugar between 140 and 199 mg/dL and diabetes was defined as a random blood sugar > 200 mg/dL.

**Kidney disease:** the study nurse collected a urine specimen from all participants and tested the specimen for proteinuria as a marker of kidney disease using the Combur-Test urine reagent strip (Roche Diagnostics, Indianapolis, Indiana, USA). Proteinuria was defined as having at least 1+ protein on a urine reagent strip. Kidney disease was defined as the presence of proteinuria as described in the Kidney Disease Improving Global Outcomes Working Group guidelines and in other studies in similar settings [21, 22].

**Overweight, obesity, and central obesity:** weight was measured using a digital scale and recorded in kilograms to the nearest 0.01 kg by the study nurse. The scale was calibrated monthly. Height was measured by the study nurse with a stadiometer and recorded in centimetres to the nearest 0.1 cm. All participants were asked to remove their shoes when measuring height and weight. Body mass index (BMI) was calculated by dividing weight in kilograms by height in meters squared (kg/m^2^). Overweight was defined as a BMI greater than 25 kg/m^2^ and obesity was defined as a BMI greater than 30 kg/m^2^. Central obesity was defined as having a waist circumference > 94 cm in men or > 80 cm in women, per the World Health Organization criteria [23]. Central obesity was included in obesity assessments as it is a known risk factor for cardiovascular mortality, independent of BMI [24].

**Self-reported medical comorbidities:** the KAP survey also included questions about previously diagnosed chronic medical conditions including diabetes mellitus, kidney disease, high cholesterol, stroke, heart attack and heart failure. Participants who responded yes to the question, ‘Prior to today, have you ever been told you have diabetes, kidney disease, high cholesterol, stroke, heart attack, or heart failure?’ were identified as being aware of a prior medical comorbidity. Participants who identified as being aware of a prior medical comorbidity were also asked if they had ever been prescribed medication for that condition, and were asked to provide the name and duration of treatment if known.

**Demographics and HIV-measures:** all participants were asked questions about education, income and marital status. In addition, a chart review was conducted to determine HIV characteristics, including date of HIV diagnosis, date of antiretroviral therapy (ART) initiation, most recent viral load, most recent CD4, CD4 nadir and current ART regimen.

**Data management and analysis:** data were collected on paper forms and entered into a REDCap database by a member of the research team. Approximately 20% of all data were verified after electronic data entry by an independent reviewer to ensure accuracy. Data were analysed using STATA version 16.0 (STATA Corp., College Station, TX). Descriptive statistics were used to describe sample demographics, characteristics, and results from the KAP survey. To examine prevalence of comorbidities, the proportion of all participants who met study criteria for obesity, diabetes mellitus and kidney disease was determined. Continuous variables that were normally distributed were expressed using mean and standard deviation (SD), and those that were not normally distributed were expressed using median and interquartile range (IQR). Categorical variables were expressed as frequencies. As this was an observational, descriptive study, no sample size calculations were performed in advance, and sample size was dictated by the ability to enrol eligible participants within the study enrolment period.

### Ethics

Study procedures were approved by the Duke Health Institutional Review Board (Pro00091126), the Kilimanjaro Christian Medical University College Ethics Committee (No. 2265), the Tanzania National Institute for Medical Research Ethics Coordinating Committee (NIMR/HQ/R.8a/Vol. IX/2779), and with the 1964 Helsinki declaration and its later amendments of comparable ethical standards. The informed consent was read out loud in Swahili by the study nurse to ensure understanding, and all participants provided written informed consent in Swahili prior to enrolment. Participants with abnormal findings received brief counselling about the results from a physician researcher from the study team, hypertension educational pamphlets, and instruction to follow up with a medical provider the same day for further evaluation and management. Participants were reimbursed approximately 5,000 to 7,000 Tanzanian shillings (approximately $2.22 to $3.11 in 2018 USD) for the cost of their time and transportation.

## Results

**Demographics and HIV-related characteristics:** a total of 105 patients met study criteria for hypertension and were eligible for study inclusion, of whom 91 (86.7%) consented to participate. Characteristics and hypertension risk factors of participants are shown in Table 1. Most participants were female (n=75, 82.4%) and mean age (SD) was 51.1 (11.2) years. While the majority (n=69, 86.3%) had controlled HIV with a suppressed viral load < 200 copies/mL, no participant met study criteria for blood pressure control.

**Table 1:** characteristics and traditional risk factors of hypertensive participants attending an HIV treatment centre, northern Tanzania, 2018 (n=91)

Demographics	
Age (years), mean (SD)	51.1 (11.2)
Sex (female), n (%)	75 (82.4%)
Education, n=90, n (%)	
Primary school or less	75 (83.3%)
Secondary school or higher	15 (16.7%)
Income per month (USD), median (IQR)	35 (17 - 67)
**HIV-related characteristics**	
HIV duration (months), n=85, median (IQR)	46 (18 - 82)
ART duration (months), median (IQR)	41 (18 - 72)
Current ART, n (%)	
TDF, 3TC, EFV	72 (79.1%)
AZT, 3TC, NVP	9 (9.9%)
TDF, FTC, EFV	5 (5.5%)
Other	5 (5.5%)
CD4 current (cells/mm^3^), n=90, median (IQR)	454 (348 - 603)
CD4 nadir (cells/mm^3^), n=90, median (IQR)	240 (117 - 382)
HIV RNA level (copies/mL), n=80, n (%)	
0 - 199	69 (86.3%)
200 - 999	2 (2.5%)
>1000	9 (11.3%)
**Traditional hypertension risk factors**	
Family history of hypertension (yes), n=90, n (%)	36 (40.0%)
Daily fruit intake (yes), n (%)	27 (29.7%)
Daily vegetable intake (yes), n (%)	36 (39.6%)
Always add salt while cooking (yes), n (%)	76 (83.5%)
Vigorous or moderate intensity physical activity (yes), n (%)	78 (86.7%)
Smoking or tobacco use history, n (%)	
Never	73 (80.2%)
Former	13 (14.3%)
Current	5 (5.5%)
Alcohol use, n (%)	
Never	18 (19.8%)
Former	35 (38.5%)
Current	38 (41.8%)
Current high risk, n=38	13 (34.2%)

Sample size is 91 participants unless otherwise stated. Abbreviations: SD: standard deviation; USD: United States dollar; IQR: interquartile range; ART: antiretroviral therapy; TDF: tenofovir disoproxil fumarate; 3TC: lamivudine; EFV: efavirenz; AZT: zidovudine; NVP: nevirapine; FTC: emtricitabine; BMI: body mass index

**Traditional hypertension risk factors:** a total of 36 (40%) participants reported a family history of hypertension, approximately two-thirds did not eat fruits or vegetables daily, and 76 (83.5%) always used salt while cooking. Close to half of participants were current alcohol drinkers (n=38, 41.8%), and among those who reported current alcohol use, 13 (34.2%) met criteria for high-risk alcohol use.

**Medical comorbidities and ASCVD risk**: Table 2 summarizes medical comorbidities and cardiovascular event risk identified by study criteria. Based on study criteria, the prevalence of prediabetes, diabetes and kidney disease was 18.7%, 8.8% and 28.6%, respectively. Many participants were classified as overweight (n=39, 42.9%) or obese (n=27, 29.7%), and most (n=78, 86.7%) met criteria for central obesity. The median calculated 10-year ASCVD risk (IQR) among all participants was 8.1% (4.3% - 15.9%). A total of 25 (33.8%) participants were categorized as intermediate risk and 14 (18.9%) were categorized as high ASCVD risk. All participants who met criteria for diabetes mellitus were categorized as either intermediate or high ASCVD risk. Of the 8 participants who met study criteria for diabetes mellitus, 3 (37.5%) were aware of their diagnosis and none reported current use of anti-glycaemic medication. No participant who met study criteria for kidney disease was aware of their diagnosis. A total of 2 (2.2%) participants reported knowledge of a previous stroke, one (1.1%) reported a history of high cholesterol, and none reported a history of previous heart attack or heart failure. No participant reported current use of statin therapy.

**Table 2:** medical comorbidities and ASCVD risk among hypertensive participants attending an HIV treatment centre, northern Tanzania, 2018 (n = 91)

Medical comorbidities and ASCVD risk identified by study criteria	
Diabetes mellitus, n (%)	
None	58 (63.7%)
Pre-diabetes	17 (18.7%)
Diabetes	8 (8.7%)
Kidney disease (proteinuria present), n (%)	26 (28.6%)
BMI categorization, n (%)	
Underweight	2 (2.2%)
Normal weight	23 (25.3%)
Overweight	39 (42.9%)
Obese	27 (29.7%)
Waist circumference (cm), mean (SD), n=90	
Men	97.7 (8.1)
Women	93.5 (10.2)
Central obesity present (yes), n=90, n (%)	78 (86.7%)
10-year ASCVD risk, n=74, median (IQR)	8.1% (4.3% - 15.9%)
10-year ASCVD risk categorization, n=74, n (%)	
Low risk < 5.0%	22 (29.7%)
Borderline risk 5.0% - 7.4%	13 (17.6%)
Intermediate risk 7.5% - 19.9%	25 (33.8%)
High risk > 20.0%	14 (18.9%)

Sample size is 91 participants unless otherwise stated. Abbreviations: ASCVD: atherosclerotic cardiovascular disease; BMI: body mass index; SD: standard deviation; IQR: interquartile range

**Figure 1:**
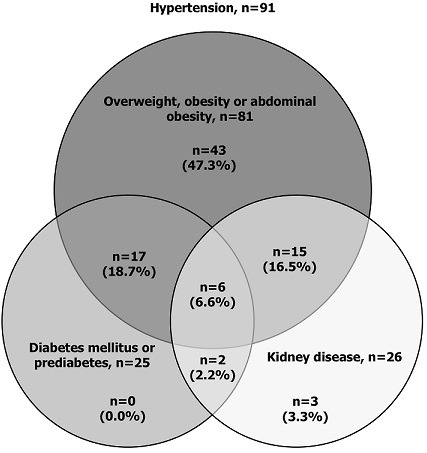
overlap of medical comorbidities including prediabetes or diabetes mellitus, overweight, obesity or central obesity, and kidney disease among hypertensive participants attending an HIV treatment centre, northern Tanzania, 2018 (n=91)

**Overlap of medical comorbidities:** overlap of medical comorbidities, including 1) hypertension, 2) diabetes mellitus or prediabetes, 3) kidney disease, and 4) overweight, obesity or central obesity is shown in Figure 1. Among the 91 hypertensive participants, only 5 (5.5%) had isolated hypertension. A total of 46 (50.5%) participants had 2 medical comorbidities, 34 (37.4%) had 3 comorbidities, and 6 (6.6%) had 4 comorbidities. The median calculated ASCVD risk (IQR) among participants with 4 comorbidities was 19.2% (14.7% - 22.2%), 7.5% (4.5% - 22.9%) among those with 3 comorbidities, and 8.1% (4.3% - 12.2%) among those with 2 comorbidities. Among participants with isolated hypertension, median calculated ASCVD risk (IQR) was 1.2% (0.8% - 1.6%).

**Hypertension Knowledge**: when asked ‘What is hypertension?’, only 3 (3.3%) participants were able to define hypertension correctly. The majority of participants defined hypertension as their perception of the symptom or the cause. Participants provided responses like ‘(Hypertension) is *when you see something that shocks you and your heart beats very fast and you fall down ’*or ‘(Hypertension) is *caused by thinking too much, or it is thinking too much’*. Almost all participants (n=86, 95.6%) believed that stress and having worries was the most common cause of hypertension. In addition, all but one participant believed that decreasing worries was an effective way to treat hypertension. The majority of respondents recognized that dietary changes, exercise, weight loss, and medications were effective ways to treat hypertension. Approximately half of participants believed that using condoms, taking antibiotics, and drinking tea were effective methods to reduce blood pressure. Table 3 represents participants responses to questions regarding hypertension knowledge.

**Table 3:** responses to questions regarding hypertension knowledge among patients attending an HIV treatment centre, northern Tanzania, 2018 (n = 91)

Questions regarding hypertension knowledge	Yes, ^a^ n (%)
**Can the following things cause high blood pressure?**	
Stress	90 (98.9%)
Weight	82 (90.1%)
Salt	79 (86.8%)
Family history	72 (79.1%)
Aging	64 (70.3%)
Alcohol	52 (57.1%)
Smoking	49 (53.9%)
**Does high blood pressure cause problems to the following body parts?**	
Eyes	81 (89.0%)
Ears	64 (70.3%)
Brain	53 (58.2%)
Heart	52 (57.1%)
Kidney	29 (31.9%)
Stomach	23 (25.3%)
**Are the following treatments or activities effective ways to reduce blood pressure?**	
Decreasing worries	87 (95.6%)
Dietary changes	84 (92.4%)
Exercising	83 (91.3%)
Weight loss	81 (89.0%)
Medications	76 (83.5%)
Using condoms	53 (58.3%)
Antibiotics	49 (53.9%)
Drinking tea	40 (44.0%)

a Other responses included “No” and “I don´t know”

**Hypertension attitudes:** most participants (n=58, 63.7%) reported never having received information about high blood pressure from a medical provider. However, almost all agreed that they needed more information about hypertension, (n=88, 96.7%). Most respondents (n=76, 83.5%) believed that hypertension was a problem in the Kilimanjaro Region, and over half of all participants, (n=50, 54.9%), worried that hypertension would negatively impact their future. Although the majority of participants (n=64, 70.3%) believed that obesity was bad for one´s health, over half (n=53, 58.9%) did not view their own weight as unhealthy and did not think they needed to reduce the amount of food they consumed (n=64, 70.3%).

**Hypertension practices:** most participants (n=89, 97.8%), expressed a preference towards seeking care at a health facility for their hypertension, as opposed to seeking care from a traditional healer (n=14, 15.4%), or using herbal or natural treatment (n=19, 20.9%). The majority of participants (n=79, 87.8%) reported they would be willing to take a medication every day for the rest of their life to treat hypertension and also expressed willingness to make significant lifestyle changes in order to reduce their blood pressure, including changing their diet (n=83, 92.2%), exercising (n=83, 91.2%), abstaining from alcohol (n=65, 89.0%), stopping smoking (n=15, 83.3%), and losing weight (n=64, 70.3%).

## Discussion

We identified a high burden of medical comorbidity and cardiovascular risk among a cohort of hypertensive patients enrolled in HIV care in northern Tanzania. However, awareness of these comorbidities and risks was low. While awareness was suboptimal, willingness to engage in the medical system and markers of care engagement were quite high. Our findings suggest that despite high levels of HIV care engagement, PLHIV are not receiving adequate screening or treatment for NCDs, and that an effective HIV health system has not provided similar benefits for other chronic conditions in this setting. High quality, evidence-based initiatives that seek to improve hypertension and NCD screening and awareness among PLHIV are urgently needed in SSA.

Similar to other studies in SSA, we found a high prevalence of traditional risk factors for hypertension and cardiovascular disease as well as a high prevalence of other chronic conditions often associated with hypertension, including diabetes mellitus, renal dysfunction and obesity [10, 11, 25]. Even more concerning, most participants (95% per our study criteria) had multiple medical comorbidities, but were unaware of their comorbid conditions and, thus, were not receiving adequate treatment. In addition, more than half of all participants were at intermediate or high risk for a serious cardiovascular event, and the risk of developing such an event appeared to increase substantially with multiple comorbidities. ASCVD risk approached 20% among those with 4 medical comorbidities. The AHA and ACC now recognize HIV as an ASCVD risk enhancing factor, and recommend consideration of statin therapy for those with borderline, intermediate or high ASCVD risk in order to reduce cardiovascular-related morbidity and mortality in this high risk population group [20]. Given that no participant in our study reported statin use, this is problematic. PLHIV are more than two-fold likely to develop cardiovascular disease, including myocardial infarction and stroke, compared to the general population [26], and now may be more likely to die from NCDs, including cardiovascular disease, rather than HIV itself [27]. Furthermore, in face of the COVID-19 global pandemic, the implication of having multiple comorbidities and increased cardiovascular risk has detrimental clinical consequences for both PLHIV and the general population. Hypertension is the most prevalent comorbidity among hospitalized patients with COVID-19 [28]. Furthermore, hypertensive individuals may have more than a twofold increased risk of developing serious COVID-19 related complications compared to individuals without hypertension [29]. Addressing hypertension and its associated comorbidities has never been more critical, and increasing awareness of cardiovascular risks and chronic diseases, beyond HIV, by improving screening and diagnosis is urgently needed to improve outcomes among PLHIV.

Levels of hypertension knowledge were mixed. Participants could not define hypertension, but had some understanding of hypertension causes, complications and treatment. In addition, most participants believed that hypertension is largely driven by psychosocial factors, such as stress, rather than by traditional risk factors, and that stress reduction was the most effective way to control blood pressure. While stress may be associated with adverse health outcomes [30], it is not considered a major aetiology of hypertension and stress reduction is not effective primary hypertension management [31,32]. Despite suboptimal levels of knowledge, participants displayed positive healthcare seeking attitudes, and expressed willingness to engage in the medical system and take medications regularly for their hypertension. Furthermore, although no participant had controlled blood pressure, almost all were adherent to their ART and virologically suppressed. Therefore, it is possible that poor hypertension outcomes are not a direct consequence of poor health seeking behaviours and disengagement from the medical system, but rather due to limitations in hypertension education and awareness. By improving hypertension knowledge through educational interventions one may also improve blood pressure control [33, 34]. Due to concentrated efforts to increase HIV awareness in SSA, most people have been exposed to information about HIV outside of the clinical setting and are knowledgeable about HIV even before initiating HIV care [35]. HIV educational campaigns have led to improvements throughout the HIV continuum of care [36]. In contrast, levels of knowledge and awareness for NCDs, including cardiovascular disease in SSA are low [37]. In recognition of this emerging hypertension and cardiovascular disease epidemic in SSA, the Pan-African Society of Cardiology (PASCAR) recently prioritized hypertension as the highest area of concern in the region. Moreover, PASCAR identified limitations in patient education, false health beliefs and poor awareness of hypertension as the major patient-related roadblocks to hypertension control. A 10-point action plan was created to address these roadblocks and improve hypertension outcomes, including investing in population level interventions such as health promotional campaigns [38]. With regional and national support, health promotional and educational campaigns can be utilized to improve hypertension knowledge, awareness and diagnoses on a broader scale. Health promotional campaigns, such as mass media educational campaigns, that target hypertension have shown some promise in reducing systolic and diastolic blood pressure and hypertension risk factors [39]. The success of community and national HIV educational campaigns in SSA could be leveraged to further target hypertension and other chronic diseases on a much larger scale to address the growing burden of NCDs among PLHIV.

Universally, no participant in our study had controlled blood pressure. In contrast, almost all had well controlled HIV with documented virologic suppression, suggesting that patients who are enrolled in HIV care routinely attend HIV clinic and are adherent to their HIV therapy [40]. As a consequence, HIV clinics may represent an available and successful healthcare platform that could be mobilized to improve hypertension and NCD outcomes in this population. Integrating routine hypertension, NCD, and cardiovascular risk screening into the HIV clinical setting may be an important approach to mitigate the growing burden of chronic disease in patients living with HIV [11, 41, 42]. Several studies in SSA have shown that integration of NCD and HIV care may be feasible, acceptable and efficacious [43-45]. Furthermore, the integration of hypertension detection, treatment and control within existing health services, such as HIV health systems has been acknowledged as a critical component of hypertension management and is part of PASCAR´s 10 point action plan for successful blood pressure control [38]. The tremendous progress made towards ending the HIV epidemic in SSA is now threatened by an increased prevalence of chronic disease outside of HIV that are associated with increased cardiovascular mortality. SSA may be faced with a dual epidemic and the utilization of robust, current care models to manage this emerging challenge is of critical importance.

This study had several limitations. First, social desirability bias may have influenced participants´ responses in the KAP survey. Participants may not have been completely forthcoming when asked questions regarding their health seeking attitudes and behaviours. Second, our recruitment strategy only included patients who were enrolled in HIV care in an urban area of northern Tanzania; therefore, our findings may not be generalizable to patients with HIV in other settings in SSA and patients with HIV who are disengaged in care, particularly younger men. Third, we used the presence of protein in the urine at a single time point as a marker for kidney disease and renal dysfunction. As such, the prevalence of kidney disease may have been overestimated in our study. Future studies measuring albumin in the urine and blood urea nitrogen, creatinine, glomerular filtration rate and creatinine clearance over time are needed to assess chronic renal disease among patients with HIV in SSA. Fourth, we used a single fasting blood sugar as diagnostic criteria for diabetes mellitus. More accurate diagnostic testing in future studies should either include a haemoglobin A1c or 2 separate fasting blood sugar measurements. In addition, we used the Pooled Cohort Equations to categorize risk of cardiovascular events. However, ASCVD risk prediction scores have not been validated in populations in SSA and should be interpreted with caution. Further studies investigating optimal cardiovascular risk prediction tools among populations in SSA are needed [46]. Lastly, as there are no existing locally validated KAP surveys for hypertension in Tanzania or SSA, we created a survey instrument and included some questions adapted from other KAP surveys that were validated in other settings and other languages. As such, the psychometric properties of our survey instrument are unknown and results should be interpreted with caution.

## Conclusion

We found a high prevalence of medical comorbidity and cardiovascular risk among a cohort of hypertensive patients enrolled in HIV care. Despite this high burden of disease, awareness of chronic medical conditions, beyond HIV, was alarming low. Given the increased risk of serious cardiovascular events associated with medical comorbidity, it is imperative to develop interventions that seek to improve NCD clinical outcomes among PLHIV. The integration of routine NCD and ASCVD risk screening in the HIV clinical setting, in combination with large-scale, culturally salient, educational campaigns, have the potential to significantly impact chronic disease outcomes in this high risk, growing, and aging population.

### What is known about this topic

- *People living with HIV have a high prevalence of hypertension with rates approaching 30% in some settings, which may significantly increase the risk of cardiovascular disease and other associated chronic conditions;*
- *Despite this high prevalence, rates of screening, awareness, treatment and control of hypertension is low*.

### What this study adds

- *Hypertensive people living with HIV have multiple medical comorbidities and have high risk for cardiovascular disease;*
- *Despite this high burden of disease, awareness, knowledge, and treatment of these conditions are suboptimal; yet, willingness to engage in the health system and markers of care engagement are high;*
- *Our results support the need for integration of non-communicable disease screening in the HIV clinical setting, as well as the expansion of educational campaigns to improve clinical outcomes among people living with HIV in sub-Saharan Africa*.
